# Colitis as the Initial Presentation of Eosinophilic Granulomatosis with Polyangiitis

**DOI:** 10.1155/2023/6620826

**Published:** 2023-10-09

**Authors:** Sharika Gopakumar Menon, Steven Hugenberg, Ahmad M. Alkashash, Jingmei Lin, Arya M. Iranmanesh

**Affiliations:** ^1^Department of Medicine, Rheumatology Division, Indiana University School of Medicine, Indianapolis, Indiana, USA; ^2^Department of Pathology, Indiana University School of Medicine, Indianapolis, Indiana, USA; ^3^Department of Radiology, Indiana University School of Medicine, Indianapolis, Indiana, USA

## Abstract

A male patient in his early sixties with recurrent diarrhea was transferred to our hospital. The patient did not have any pulmonary or upper respiratory symptoms. He was noted to have peripheral eosinophilia. Further workup revealed a negative antineutrophilic cytoplasmic antibody titer but a positive myeloperoxidase antibody and positive proteinase 3 antibodies. A colon biopsy also revealed eosinophilic-rich granulomas in the mucosa, confirming a diagnosis of eosinophilic granulomatosis with polyangiitis. On cardiac imaging, eosinophilic myocarditis was also discovered. To treat active severe EGPA, the patient received high-dose corticosteroids and intravenous cyclophosphamide. The occurrence of gastrointestinal involvement as an initial manifestation of eosinophilic granulomatosis with polyangiitis is infrequent, emphasizing the significance of its recognition. This case underscores the importance of identifying and diagnosing such atypical presentations to facilitate timely and appropriate management.

## 1. Introduction

Eosinophilic granulomatosis with polyangiitis (EGPA), formerly known as Churg–Strauss syndrome, is a rare vasculitis involving small and medium-sized vessels. Peripheral eosinophilia, multiorgan eosinophilic infiltrates, and necrotizing granulomas are pathognomonic of the condition [1–3]. Most cases present with asthma, sinus disease, pulmonary disease, and neuropathy [1]. Involvement of the heart, lungs, gastrointestinal tract, kidneys, and central nervous system may be seen. Approximately 30% of cases are associated with gastrointestinal involvement. Only about 40% of EGPA cases are known to have ANCA positivity. An increased risk of glomerulonephritis and neurological manifestations have been noted in patients with ANCA positivity. Characteristic histopathologic findings include eosinophilic infiltration, necrosis, giant cell vasculitis, and perivascular necrotizing granuloma [4–6].

## 2. Case Presentation

A 61-year-old male patient with recurrent *Clostridium difficile* (*C. difficile*) colitis with non-blood-stained diarrhea was transferred to our hospital for a possible fecal microbiota transplant. The patient was initially diagnosed with C. difficile infection after cholecystectomy and treated with oral vancomycin and daptomycin without improvement. He continued to have diarrhea, requiring admission to an outside hospital and treatment with more antibiotics, including fidaxomicin. He had leukocytosis (white blood cell count of 23000), lactic acidosis, and acute kidney injury. Blood cultures were negative for microbial growth, and a computerized tomography scan of the abdomen revealed no signs of colitis. The medical team treated the patient with oral vancomycin and intravenous metronidazole. However, the patient continued to experience diarrhea, so they transferred him to our hospital.

Upon presentation, the patient continued to experience episodes of diarrhea. He did not have a history of asthma or sinus disease, although he did have chronic obstructive pulmonary disease. The examination was significant for a mild weakness of dorsiflexion on the right side, a chronic problem for the patient. We did not find any significant peripheral edema or any increased work of breathing.

### 2.1. Investigations

Laboratory testing revealed 48% eosinophils and an absolute eosinophil count of 10.8 k/mm^3^. The patient underwent a repeat C. difficile toxin test, which was negative. The stool tests for bacteria, viruses, ova, parasites, and serum Strongyloides IgG were negative. BCR-ABL and PDGFR tests were negative, and a bone marrow biopsy revealed hypercellular marrow with trilinear hematopoiesis and significant eosinophilia. The ANCA panel test revealed a normal ANCA titer (<1 : 20), an elevated myeloperoxidase IgG of 25 (normal 0–19), and a proteinase 3 IgG of 186 (normal 0–19). The patient's urinalysis was normal.

**Figure 1:**
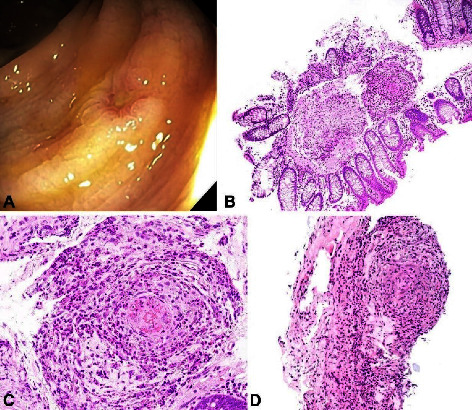
Eosinophilic granulomatosis with polyangiitis. (A) Area of patchy erythema and decreased mucosal vascularity in the rectum seen on colonoscopy. (B) Colon mucosal biopsies: low power magnification showed eosinophilic necrotizing granulomatous vasculitis involving the submucosal vessel (×40). (C, D) High-power magnification showed a dense eosinophilic infiltrate destroying the vessel wall, a classic presentation of granulomatous vasculitis (×400).

The colonoscopy revealed normal appearance appearance of the signoid, descending, and transverse colon with mild erythematous and vascular patterns in the rectum. Multiple random biopsies of the colon revealed necrotizing granulomatous vasculitis of small and medium-sized arteries, dense eosinophilic infiltration, and granulomas (Figure 1)

**Figure 2:**
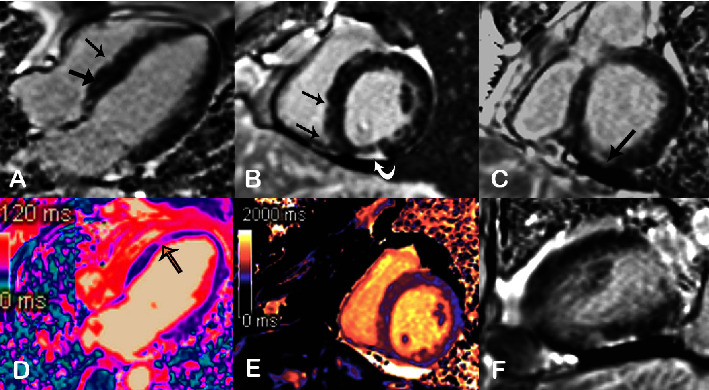
Suspected acute eosinophilic myocarditis. (A, B, C, F) Multiplanar late gadolinium enhancement (LGE) imaging is demonstrated in the 4-chamber (A), short-axis (B, C), and 2-chamber long-axis (F) planes. Unlike most contrast-enhanced sequences routinely utilized in both CT and MRI, these images are obtained in 5–15 minutes following intravenous administration of the contrast agent (gadolinium, allowing for washout of contrast from not only the intravascular space but also from most soft tissues (including normal/viable myocardium)). These cardiac-specific sequences further utilize an “inversion” radio frequency pulse before image acquisition to further “null” (decrease/darken) the signal from the myocardium. The resultant image then depicts normal myocardium as a nulled low signal (black) and acute myocardial necrosis or scar regions as a relatively high signal (gray/white). (A) In a four-chamber LGE image, the thick black arrow points to a viable, appropriately “nulled” septal myocardium. In contrast, the adjacent thin black arrow points to one of the several focal regions of epicardial hyperenhancement involving the septum. (B) Correlative short-axis findings of patchy, predominantly epicardial enhancement resulting in an irregular appearance of the septal epicardium (black arrows) and adjacent confluent hyperenhancement of the more severely involved inferior epicardium (curved white arrow). (C) A short-axis LGE view reveals additional near transmural involvement of the basal inferior wall. A color “map” plotting specific T2 and T1 values at each voxel is depicted in (D, E), respectively. Increased signal values (appearing as shades of purple in (D) and orange/yellow in (E)) suggest disease involvement/myocardial edema. The yellow arrow in (D), a two-chamber long-axis T2 map, points to the focal near transmural edema of the LV septum. (D) In a short-axis T1 map, multiple regions of abnormal predominantly epicardial signal correlate with/confirm the suspected multifocal myocardial necrosis and scar (hyperenhancement) depicted in LGE images (A, B, C, F).

Due to the cardiac involvement often seen in EGPA and the high mortality associated with this, an echocardiogram (ECHO) was performed, which revealed moderately to severely depressed left ventricular (LV) systolic function (ejection fraction (LVEF) 36%) with global hypokinesis and regions of more focal wall motion abnormality involving both left anterior descending (LAD) and right coronary artery (RCA) territories. Further evaluation with cardiac magnetic resonance imaging (cMRI) was performed to further define the etiology of left ventricular dysfunction. Although dynamic cine MR imaging again demonstrated LV systolic dysfunction with associated regions of focal wall motion abnormality, late gadolinium enhancement imaging appeared inconsistent with the sequela of coronary artery disease. Specifically, multifocal regions of delayed myocardial enhancement (correlated with the myocardial scar and/or acute myocardial necrosis) were noted, predominantly involving the epicardium and midmyocardium of the basal to midventricular inferior and septal walls (Figure 2). The “patchy” hyperenhancement pattern involving multiple coronary territories, and largely sparing the more poorly perfused subendocardium, was felt to be most suggestive of eosinophilic myocarditis in the setting of peripheral eosinophilia and suspected EGPA. Electrodiagnostic testing or imaging to evaluate the weakness of dorsiflexion was not performed during the patient's hospitalization.

### 2.2. Differential Diagnosis

The patient had recurrent diarrhea, which was concerning for recurrent Clostridium difficile infection with possible failure of response to antibiotic treatment. The patient had to repeat Clostridium toxin testing at our facility, which was negative. He also underwent a colonoscopy, which showed no signs of pseudomembranous colitis typical of C. difficile infection. Our patient had profound peripheral eosinophilia, and it was imperative to rule out causes for this, including parasitic infections and hypereosinophilic syndrome (HES). Stool testing for ova and parasites was negative, and we also performed serum Strongyloides testing, although the patient had no travel history to an endemic area. HES can be primary or secondary, which is also known as reactive. Primary HES can be due to myeloproliferative disorders with myeloid or lymphoid neoplasm. We performed testing for both BCR-ABL and PDGFR, which were negative; hence, a myeloproliferative disorder was thought to be unlikely. With colonic pathology showing eosinophilic granulomas, this was considered pathognomonic of EGPA, and therefore, a definite diagnosis was made. Although granulomatosis with polyangiitis (GPA) remains a potential consideration among ANCA-positive patients with vasculitis, extensive peripheral eosinophilia, colonic eosinophilic granulomas, and the presence of cardiomyopathy collectively provide stronger evidence for a more conclusive diagnosis of eosinophilic granulomatosis with polyangiitis (EGPA). Furthermore, it is noteworthy that GPA is predominantly linked to manifestations involving the ear, nose, and throat (ENT) and pulmonary manifestations. In contrast, our patient's clinical presentation did not encompass any ENT-related or pulmonary findings.

### 2.3. Treatment

Our patient had a five-factor score of two for cardiac involvement and absence of ear, nose, and throat involvement. Thus, the patient's disease was categorized as severe EGPA. Gastrointestinal involvement in the five-factor score is defined as bleeding, perforation, or pancreatitis. Our patient did not have these. Therefore, we followed the recommendations of the EGPA consensus task force and initiated high-dose prednisone and cyclophosphamide treatment. Based on the recommendations of the EGPA consensus task force, the patient received cyclophosphamide 15 mg/kg intravenously (IV) every two weeks for three doses and three additional infusions of 15 mg/kg every three weeks [7]. The first dose was administered in the hospital.

### 2.4. Outcome and Follow-Up

He completed six cycles of cyclophosphamide, and prednisone was tapered off. Unfortunately, the patient died at home from presumed cardiac arrest as per family, although this is not confirmed. No autopsy was performed.

## 3. Discussion

EGPA is an ANCA-associated vasculitis that typically presents with a prodrome of fever, malaise, and respiratory symptoms. The involvement of the upper and lower respiratory tract is common. Asthma, allergic rhinitis, nasal polyps, and sinusitis are common manifestations of the disease [3, 8]. The disease primarily affects individuals between the ages of 35 and 50, without specific gender or ethnic predilection [9, 10]. Some investigators hypothesize an association between some medications such as leukotriene receptor antagonists. However, this is likely due to unmasking the underlying disease rather than directly causing EGPA [11]. Several studies showed a positive association between EGPA and HLA-DRB1 and HLA-DRB4 gene positivity [12].

The pathogenesis of EGPA has yet to be completely understood. It is considered primarily T-cell-mediated vascular injury. CD4 T lymphocytes secrete gamma interferon, which promotes granulomatous inflammation [13, 14]. Interleukins IL-4, IL-5, and IL-13 activate eosinophils, thus releasing proteins that cause damage to endothelial cells. This damage leads to the release of eotaxin-3 from endothelial cells, which attracts more eosinophils [13, 14].

Lung involvement in EGPA is extensive, with an overwhelming majority, exceeding 90%, exhibiting asthma during the initial presentation. Asthma usually predates the development of other systemic manifestations of EGPA by almost a decade. Dyspnea on exertion and productive cough are commonly observed as presenting symptoms in patients with EGPA-related lung involvement. Although relatively rare, respiratory failure can be an initial presenting feature in a small subset of cases. Radiographically, various abnormalities are detected in EGPA-related lung involvement, including ground glass attenuation, consolidation, nodules, and pleural effusion. Alveolar hemorrhage may be noted in a minority of cases [2, 15, 16]. A small subset of patients, approximately 20%, may have renal involvement, with an overall increased incidence noted in ANCA-positive patients. Rapidly progressive glomerulonephritis and acute renal failure may be observed with EGPA [15, 17]. Cardiac involvement is common and associated with an almost 50% mortality rate. Manifestations include arrhythmias, pericarditis, restrictive cardiomyopathy, eosinophilic myocarditis, and coronary vessel vasculitis. Endomyocardial disease, also called Löeffler endocarditis, may be seen in EGPA, like other hypereosinophilic disorders. Löeffler endocarditis is considered to represent the most severe form of cardiac involvement. Echocardiography and, if available, cardiac MRI are the most common diagnostic modalities employed to diagnose cardiac involvement. Delayed gadolinium enhancement is typically observed and most frequently involves the apical and midcavity segments. Myocardial biopsy may be considered [18]. The hematoxylin and eosin sections of the myocardium usually demonstrate extensive inflammatory infiltration with numerous eosinophils. Endocardial thickening, myocyte necrosis, granulomas, pericarditis, thrombi, and coronary artery vasculitis can all be noted in pathology [18].

GI involvement occurs in nearly half of the patients diagnosed with EGPA, with symptoms including abdominal pain, vomiting, and diarrhea [19]. However, GI symptoms as an initial presentation of EGPA are rare. Diagnosing EGPA in the GI tract requires high clinical suspicion since symptoms can vary from nonspecific to severe surgical abdomen. In the gastrointestinal tract, histology sections usually show expansion of the lamina propria by eosinophils and other inflammatory cells with eosinophilic infiltration of the surface epithelium. In addition, blood vessels of the lamina propria usually show eosinophilic necrotizing granulomatous vasculitis with villous blunting and mucinous metaplasia reported in cases with stomach and duodenum involvement [19, 20].

Elevated C-reactive protein and IgE levels may be noted in serum studies. Eosinophilia is usually present in more than 10% of the patients, and anti-MPO ANCA is detected in 30–60% of cases [15, 21]. ANCA titers can indicate disease activity and renal involvement, with MPO positivity associated with increased relapse rates. Conversely, ANCA-negative cases are more prone to cardiac involvement [21, 22].

To our knowledge, GI involvement as an initial presentation of EGPA is rarely reported in the literature, despite nearly half of the patients diagnosed with EGPA having GI involvement [19]. In addition, documentation of the actual pathology is seldom reported since vasculitis is rarely noted on biopsy sections due to the superficial nature of the sample [19, 20]. Our case emphasizes the importance of considering EGPA in patients with hypereosinophilia and GI symptoms. This patient had granulomas noted on biopsy, but most often, a biopsy of the intestinal mucosa reveals only eosinophilia.

The ACR/EULAR 2022 classification criteria for EGPA include obstructive airway disease, nasal polyps, mononeuritis multiplex, laboratory criteria of an eosinophil count of 1 × 10^9^/L, extravascular eosinophil predominant inflammation, and hematuria. The presence of cANCA or PR3 antibodies decreases the total score [23]. In our patient, the presence of PR3 positivity would result in a reduction of his ACR classification criteria score. However, it is important to acknowledge, as commonly observed in rheumatological cases, that the utilization of classification criteria alone is insufficient for making a definitive disease diagnosis.

Broadly, treatment guidelines are based on whether the disease is severe or nonsevere, based on end-organ damage. In active and severe EGPA cases, a combination of pulse corticosteroids and high doses of corticosteroids, along with additional immunosuppressive agents, is recommended. Cyclophosphamide and rituximab are the preferred choices of medication for severe cases. Rituximab is generally favored for ANCA-positive patients. Due to the cardiac involvement in our case, we selected cyclophosphamide as it has more substantial data supporting its use in treating this specific subset of EGPA patients. This choice was further influenced by the patient's age (male in his 60s) and the absence of fertility preservation concerns, which contributed to a more manageable side effect profile. Another concerning side effect of cyclophosphamide is cardiotoxicity, though this is thought to be dose-dependent. Our patient did not have any worsening of his symptoms and completed the induction therapy.

For nonsevere EGPA, a lower dosage of glucocorticoids can be used in conjunction with other immunosuppressive agents. These agents may include methotrexate, azathioprine, mycophenolate, rituximab, or mepolizumab. The American College of Rheumatology suggests initiating treatment with mepolizumab specifically for patients with nonsevere EGPA. Thus, we excluded it as an induction therapy option due to the severity of our patient's condition.

During the remission phase, it is advisable to continue maintenance therapy using these immunosuppressants [24].

### 3.1. Learning Points

1. Although pulmonary and sinus complaints are the most common presenting symptoms in patients with EGPA, a subset may present with GI manifestations. Therefore, the absence of active pulmonary symptoms should not make clinicians overlook the diagnosis of EGPA.
2. Our patient did not exhibit any symptoms or signs of heart failure but had reduced EF and myocardial involvement, emphasizing the critical importance of evaluating cardiac disease in EGPA even when patients are asymptomatic. This is especially important given high mortality is associated with cardiac involvement.
3. Our patient did not meet the ACR classification criteria for EGPA but has a biopsy-proven disease. Therefore, it is imperative to consider the case in its entirety.
4. Severe EGPA warrants treatment with high-dose or pulse-dose corticosteroids; cyclophosphamide is the preferred immunosuppressive agent, especially in patients with cardiac involvement.

## Data Availability

The data used to support the study are cited, and the articles referenced can be found in the manuscript.
